# Severe SARS‐CoV‐2 infection in a 32‐week pregnant woman treated with Remdesivir‐Dexamethasone combination therapy: A case report

**DOI:** 10.1002/ccr3.6241

**Published:** 2022-08-11

**Authors:** Seyed Mohammad Amin Alavi, Samaneh Bahrami, Mahin Najafian, Mina Hoori

**Affiliations:** ^1^ Student Research Committee Ahvaz Jundishapur University of Medical Sciences Ahvaz Iran; ^2^ Division of Perinatology, Department of Obstetrics and Gynecology, Imam Khomeini Hospital Ahvaz Jundishapur University of Medical Sciences Ahvaz Iran

**Keywords:** cesarean section, COVID‐19, pregnancy; premature birth, SARS‐CoV‐2

## Abstract

This study shows that remdesivir and dexamethasone combination therapy can be considered as a suitable treatment choice for pregnant women infected with COVID‐19. It is worth mentioning that more studies are required to evaluate the efficacy and side effects of remdesivir monotherapy and its combination with dexamethasone during pregnancy.

## Graphical abstract

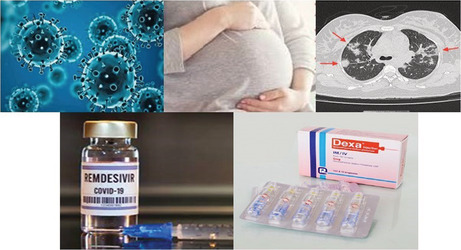

## 1 INTRODUCTION

On December 31, 2019, the Wuhan Municipal Health Commission announced the first cluster case of pneumonia in Wuhan, Hubei Province, China. The new coronavirus variant, identified by Chinese authorities, was isolated on January 7, 2020. The World Health Organization (WHO) ranked this disease as the sixth public health emergency of international concern.^1^ Coronavirus is a positive‐stranded ribonucleic acid (RNA) virus with an envelope. It is a member of the Nidovirales order and the Coronaviridae family.^2^ Coronaviruses cause respiratory and gastrointestinal infections, ranging from mild, self‐limited viruses to more severe illnesses such as viral pneumonia.^3^ Pregnancy‐related physiologic maternal adaptations are well recognized, making pregnant women more vulnerable to a more severe course of pneumonia, resulting in higher maternal and fetal morbidity and mortality.^2^, ^4^

In the present article, the researchers describe a pregnant woman infected with SARS‐CoV‐2 for whom performing early medical and surgical intervention saved both the mother and infant's lives.

## 2 CASE PRESENTATION

The patient was a 37‐year‐old Iranian singleton pregnant woman gravida 1. Gestational age was 32 weeks and 6 days. She had been previously diagnosed with hypothyroidism and then received Levothyroxine 50 μg tablet daily. Body mass index (BMI) was $30\frac{\mathrm{kg}}{m^{2}}$.

**FIGURE 1:**
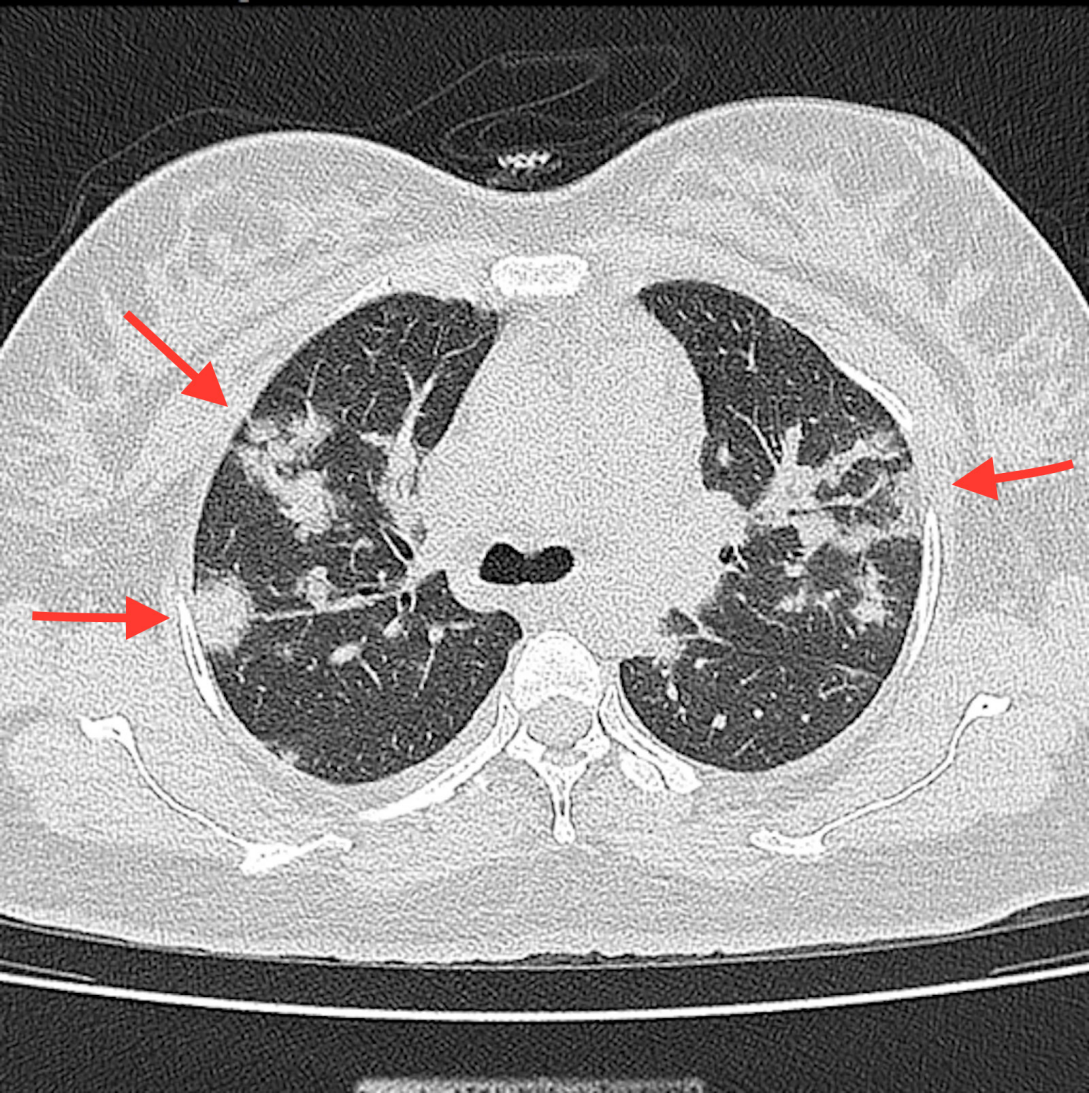
Multifocal ground‐glass opacities in low‐dose high‐resolution chest computed tomography (HRCT)

Her symptoms were dyspnea and fever, which started 2 days before admission. There was no evidence that the patient had any connection to anyone who was SARS‐CoV‐2 infected at the time (Due to the lack of evidence of exposure, the authors cannot report day 0 of infection definitely). The SARS‐CoV‐2 real‐time reverse transcriptase‐polymerase chain reaction (real‐time RT‐PCR) test was positive, so the patient was admitted to our tertiary hospital on July 13, 2021. The vital signs on arrival time were as follows: blood pressure:120/80 mmHg, pulse rate 83, respiratory rate 23, and body temperature of 38°C. Moreover, O_2_ saturation was 95% breathing ambient air and 98% with an oxygen mask at her admission. Due to the considerable involvement of the lungs as observed in low‐dose high‐resolution chest computed tomography scan (chest HRCT) with ground‐glass opacity of SARS‐CoV‐2 infection (Figure 1), she was admitted to the intensive care unit (ICU). After that, on the first day of hospitalization, and after performing the chest HRCT, a daily dose of 100 mg of remdesivir (with a loading dose of 200 mg) and 6 mg of dexamethasone every 12 h up to four doses and then 6 mg daily were administered intravenously to her, and was continued for 5 days. Enoxaparin 40 mg daily for 7 days was also administered subcutaneously for this patient. Electrocardiography (ECG) was performed, which showed normal sinus rhythm with no abnormal changes. In addition, echocardiography was done, which revealed normal cardiac function with 60% left ventricular ejection fraction (LVEF).

On the second day of hospitalization, O_2_ saturation progressively decreased (60%–70%), resulting in intubation. In addition, mechanical ventilation was also started for the patient. Due to the repeated late deceleration in cardiotocography (CTG), an emergency preterm cesarean delivery was performed. The neonate's Apgar scores were 2/10 and 6/10, so the resuscitation protocol was immediately initiated. Afterward, the neonate was admitted to the neonatal intensive care unit (NICU). Of note, the newborn's SARS‐CoV‐2 real‐time RT‐PCR was negative. The newborn was discharged after 10 days in an entirely normal condition. Moreover, the newborn did not develop any symptoms before the first evaluation after the discharge.

On the fifth day of hospitalization, the patient was extubated successfully. After *extubation*, the patient was given oxygen via a face mask and nasal cannula. Moreover, after complete recovery, the patient was discharged on the ninth day of hospitalization. O_2_ saturation at the time of discharge was noted at 99% breathing ambient air. After performing several evaluations, the mother and her newborn were found to be in normal condition.

## 3 DISCUSSION

As per recent studies, pregnant women with SARS‐CoV‐2 infection have a significantly increased risk of ICU admission and mechanical ventilation in comparison with non‐pregnant women.^5^ Preterm delivery is now the most prevalent adverse pregnancy result among hospitalized pregnant women with coronavirus infections, particularly SARS‐CoV‐2, and >90 percent of whom had pneumonia, as well.^6^ The vertical transmission of the coronavirus causing a severe acute respiratory syndrome is conceivable and occurs in a minority of patients with maternal SARS‐CoV‐2 infection in their third trimester.^7^ Real‐time RT‐PCR of our patient's newborn was reported to be negative, and the vertical transmission did not occur, fortunately.

Abnormal or indeterminate fetal heart rate tracing is one of the most common indications for cesarean delivery,^8^ and as mentioned earlier, in our case, the cesarean delivery indication was due to abnormal CTG.

Physiological changes during pregnancy could affect some of the factors of the blood tests. For example, CRP and Interleukin‐6 may be elevated during pregnancy.^9^ However, the most reliable SARS‐CoV‐2 indicators among pregnant women were the followings: the elevated C‐reactive protein (CRP), leukocytosis, and an increased neutrophil ratio.^10^ All of the above‐mentioned laboratory findings increased in the patient's tests which might be due to SARS‐CoV‐2 infection (Table 1). Although Interleukin‐6 was elevated in our patient's laboratory findings, procalcitonin was within the normal range. The literature indicates that an excessive amount of Interleukin‐6 may be seen in the third trimester of pregnancy laboratory analysis.^9^ On the contrary, some laboratory findings suggest severe SARS‐CoV‐2 infection. For example, an elevated D‐dimer level may be seen not only during pregnancy but also in SARS‐CoV‐2 infection. It should also be mentioned that a three‐ to four‐fold increase in the amount of D‐dimer is one of the poor prognosis factors of SARS‐CoV‐2 infection.^11^ Moreover, in the patient, an increased D‐dimer and lactate‐dehydrogenase (LDH), decreased absolute lymphocyte count, and extensive lung involvement in low dose HRCT (more than 50 percent) suggested that the illness had progressed to a more severe stage. Based on the aforementioned laboratory data, two differential diagnoses were ruled out. CRP and Interleukin‐6 may rise in bacterial infections.^12^; Therefore, due to this reason, further investigations were conducted to rule out bacterial infection by the blood and urine cultures. In addition, an elevated D‐dimer level is used to predict thrombosis in the lungs and lower limbs.^11^ Lower limb color doppler ultrasound and chest computed tomography (CT) angiography were conducted to rule out thrombosis in the current study because of the increased D‐dimer level. No evidence of thrombosis was found.

**TABLE 1:** Laboratory findings

Laboratory parameter	Admission	HD1	HD2	HD3	HD4	HD5	HD6	HD7
Leukocyte ×1000	11.9	12.1	11.3	12.2	11.3	11.2	11.5	12.6
Lymphocyte (%)	6.7	7	10	14.3	9.5	15.5	41	52
Neutrophils (%)	77.6	73.1	81.5	69	86.2	76.7	52	38.6
Platelets ×1000	136	166	157	212	214	275	223	247
Hemoglobin (gr/dl)	9.9	10.4	9.7	9.2	10.2	9.3	10.9	11.3
CRP	Trace	ND	ND	ND	ND	ND	ND	3+
Cr (mg/dl)	0.8	0.9	0.9	1	1	0.8	0.7	0.6
Na (mEq/L)	149	143	149	150	149	141	140	139
K (mEq/dl)	3.5	4.6	4.7	4.1	4	4.1	4.5	5.2
Ca (mEq/dl)	9.5	ND	8.6	ND	8.7	7.5	ND	ND
P (mEq/dl)	3.6	ND	6.2	ND	4.4	3.6	ND	ND
Mg (mEq/dl)	ND	ND	2.1	ND	2.2	ND	ND	ND
AST (IU/L)	28	ND	39	23	29	31	ND	33
ALT(IU/L)	23	ND	35	24	16	21	ND	34
ALP(IU/L)	203	ND	170	201	236	188	ND	193
LDH (IU/L)	449	ND	ND	ND	ND	ND	ND	ND
TSH(IU/L)	1	ND	ND	ND	ND	ND	ND	ND
BS (mg/dl)	81	ND	ND	ND	ND	99	ND	73
Albumin (g/L)	3	ND	2.7	ND	3.5	3.2	ND	ND
Total Protein (g/dl)	5.5	ND	4.7	ND	6.1	5.7	ND	ND
ABG (PH)	7.4	7.18	7.28	7.39	7.42	7.46	7.45	7.43
ABG (PCO2)	38.1	51.3	53.7	34.8	41.6	48.2	48.2	39.2
ABG (PaO2)	30.2	47.3	34	53.4	40.8	34.8	34.8	62
ABG (HCO3)	24.8	18.8	24.7	21	26.8	33.9	33.9	26
D‐Dimer	2492	ND	ND	ND	ND	ND	ND	ND
Interleukin‐6	53.8	ND	ND	ND	ND	ND	ND	ND
Procalcitonin	0.05 < 0	ND	ND	ND	ND	ND	ND	ND

Abbreviations: ABG, arterial blood gas test; ALP, alkaline‐phosphatase; ALT, alanine aminotransferase; AST, aspartate aminotransferase; BS, blood sugar; Ca, calcium; Cr, creatinine; CRP, C‐reactive protein; HD, hospitalization day; K, potassium; LDH, lactate‐dehydrogenase; Mg, Magnesium; Na, sodium; ND, not determined; P, Phosphorus; TSH, thyroid‐stimulating hormone.

Remdesivir is a nucleotide analog. This prodrug is converted to an adenosine triphosphate analog intracellular, which impedes RNA polymerases in the virus.^13^ Remdesivir is the first medication approved for SARS‐CoV‐2 based on data showing that it reduces hospitalization time.^14^ Manufacturer safety data for remdesivir showed no evidence of reproductive developmental toxicity in animals at therapeutically relevant doses. Its embryonic toxicity was only reported when systemically toxic doses were given to female animals before pregnancy. [Remdesivir health care provider's fact sheet, Gilead Sciences]^15^ Pregnant women are less likely to participate in clinical trials because of the possibility of fetal toxicity. There is no reported fetal toxicity for remdisivir, even though it has been administered in pregnant women with Ebola and Marburg virus disease.^16^ Recent studies demonstrated that the administration of dexamethasone could decrease mortality in severe SARS‐CoV‐2 infected patients who receive invasive mechanical ventilation or oxygen alone.^17^

## 4 CONCLUSION

In this study, emergency preterm cesarean delivery did not aggravate the severity of SARS‐CoV‐2 pneumonia. The neonate's SARS‐CoV‐2 real‐time RT‐PCR was negative, which supports previous studies that vertical transmission only occurs in a minority of pregnant patients.

According to the findings of this investigation, remdesivir and dexamethasone combination therapy may be regarded as a suitable therapeutic option for SARS‐CoV‐2 infection, especially in pregnancy. In this regard, it is worth noting that further investigations should be conducted to determine the effectiveness and safety of remdesivir monotherapy and its combination with dexamethasone during pregnancy.

## AUTHOR CONTRIBUTIONS

The authors designed, performed the study, and wrote the manuscript together for publication.

## CONFLICT OF INTEREST

The authors have no conflict of interest to declare.

## ETHICAL APPROVAL

There was no need for the ethics committee permission for case reports in the institution where the research was carried out.

## CONSENT

Written informed consent for publication of the case report has been signed by the patient and is available upon request from the editors.

## Data Availability

The data that support the finding of this study are available upon request from the corresponding author.
